# Comparison of Point-of-Care and Highly Sensitive Laboratory Troponin Testing in Patients Suspicious of Acute Myocardial Infarction and Its Efficacy in Clinical Outcome

**DOI:** 10.1155/2022/6914979

**Published:** 2022-02-24

**Authors:** Sahand Mohammadzadeh, Nasim Matani, Neda Soleimani, Hamed Bazrafshan drissi

**Affiliations:** ^1^Department of Pathology, Shiraz Medical School, Shiraz University of Medical Sciences, Shiraz, Iran; ^2^Department of Pathology, Shiraz Transplant Center, Abu Ali Sina Hospital, Shiraz University of Medical Sciences, Shiraz, Iran; ^3^Cardiovascular Department, Shiraz University of Medical Sciences, Shiraz, Iran

## Abstract

**Background:**

The use of high-sensitivity troponin (hs-cTnI) assays is recommended in current guidelines for managing patients with acute coronary syndrome (ACS) symptoms. However, point-of-care (POC) assays are frequently used in emergency departments (EDs) to reduce turnaround time and length of stay. This study aimed to compare the results of POC-cTnI testing with those of the gold standard, automated central laboratory testing of troponin (i.e., hs-cTnI). The primary and secondary outcomes were the diagnostic performance of POC-cTnI in diagnosing acute myocardial infarction (AMI) and major adverse cardiovascular events (MACE) during 30 days, respectively.

**Materials and Methods:**

In this diagnostic accuracy study, 136 patients with suspected ACS who were referred or admitted to the Al Zahra Hospital, Shiraz, Iran, were included between March (2020) and July (2020). For the diagnosis of AMI, central laboratory cTnI levels were assessed at the time of presentation (0 hour) and reassessed at least 3 hours later. The POC-cTnI was measured at 0 hour in all patients and at 3 hours if a patient was diagnosed with AMI but had a 0-hour negative result for the POC-cTnI assay. Additionally, the 30-day follow-up period for these participants began on the day of the initial presentation to assess MACE.

**Results:**

Out of 180 patients, 136 patients (median age of 59.5 years; 57.5% male) were left for the qualitative POC-cTnI and hs-cTnI assays. In 86 (63.24%) subjects, hs-cTnI was positive (either initial or serial); however, AMI was diagnosed in 85 patients according to positivity of troponin by hs-cTnI and clinical signs and symptoms, which were diagnosed by a cardiologist. The sensitivity, specificity, and negative predictive value of 0-hour POC-cTnI were observed to be 91.76% (95% CI: 83.77–96.62%), 98.04% (95% CI: 89.55–99.95%), and 87.72% (95% CI: 77.82–93.56%), respectively. Moreover, considering both the 0-hour and 3-hour POC-cTnI, all AMI cases were correctly identified, yielding a perfect test performance result. None of the 50 patients with negative cTnI results (by 0-hour and 3-hour POC-cTnI and hs-cTnI) experienced at least one MACE.

**Conclusion:**

In this small sample-size study, a new qualitative POC-cTnI assay was statistically equal to a hs-cTnI assay in terms of diagnostic accuracy for AMI or MACE in patients with suspected myocardial infarction. The POC-cTnI was observed to be acceptable for the identification of AMI and prediction of MACE in the ED environment.

## 1. Background

Assays to measure the level of cardiac troponin (cTn) in the blood were developed in the early 1990s [1]. Early testing revealed that the presence of cTn suggested the presence of heart damage. Since then, fast cTn testing has been an essential part of the early diagnosis of acute coronary syndrome (ACS) [2].

The second most prevalent reason for emergency hospital admission is chest discomfort. However, with improved diagnostic equipment, numerous hospital admissions might be avoided, as the prevalence of ACS in individuals hospitalized on suspicion of that diagnosis is 20%. It is critical to have a quick and correct diagnosis of acute myocardial infarction (AMI) since early action can save lives [3]. The presence of circulating cardiac troponin I or T (cTnI or cTnT) above the upper reference limit is one of the criteria used to diagnose myocardial infarction [4].

Although the significance of this diagnostic tool has long been recognized, not all measurement methods are equally helpful. The need to determine cTn levels in a timely manner is emphasized in current recommendations [5]. Accordingly, clinical laboratories are under growing pressure to reduce cardiac marker turnaround time (TAT). Blood samples are taken and forwarded to a hospital's central laboratory for cTn analysis when a patient appears with symptoms suggestive of ACS. Physicians can then acquire these results in less time owing to the advent of point-of-care (POC) assays. Although a shorter TAT is encouraging, physicians should be confident that quality is not sacrificed [6].

In addition, at the time of admission, cTn measurements are indicated, and they should be repeated 3 hours later. As a result, most emergency department (ED) patients require additional testing before being released safely. This could be linked to high healthcare expenses and overcrowding in emergency rooms [7].

High-sensitivity troponin (hs-cTn) assays are recommended in current guidelines for the management of patients with ACS. It helps to diagnose patients with signs of myocardial ischemia early and reduces the rising number of EDs crowded with patients. Despite this, POC assays are frequently used in EDs because they minimize TAT and hospital length of stay (LoS). The POC assays have been chastised for failing to meet the specified coefficient of variation of 10% at the 99th percentile upper reference limit. It is uncertain whether using traditional cut-offs versus using POC impacts overall diagnostic performance and, as a result, clinical patient management [7, 8].

The POC troponin (POC-cTn) assays fit into this early decision-making process and have recently received great attention in the emergency and cardiology literature [9]. POC assays, defined as laboratory testing close to a patient's location with quick findings, have received much interest in the ED. The problem is that current tests are insufficiently sensitive to rule-out ACS adequately. For the purposes of saving lives, producing quick and valid results, and saving money, ED cTn testing should be sensitive and dependable. At present, the gold standard in troponin assessment is automated laboratory data [10].

Because POC systems are becoming more popular and advantageous in TAT, the verification of the acquired results is vital [11]. The current study aimed to compare the results of the Instant-View POC-cTnI qualitative assay to those of the gold standard and automated central laboratory testing of troponin (i.e., hs-cTnI assay) in patients presenting to an ED with ACS symptoms and evaluate its efficacy for 30-day major adverse cardiovascular events (MACE). We also want to determine if the POC TnI repeat test after 3 hours has a higher sensitivity or specificity for detecting or ruling out ACS in patients with chest discomfort than the first sample.

## 2. Materials and Methods

### 2.1. Study Designs and Setting

This diagnostic accuracy study included 136 patients with suspected ACS who were referred or admitted to the Al Zahra Hospital, Shiraz, Iran, between March (2020) and July (2020). Patients should have blood extracted for POC-cTnI and hs-cTnI assays simultaneously without delay when they arrive in the ED (0 hour), with written informed consent obtained later. These measurements were assessed at least 3 hours later.

### 2.2. Inclusion Criteria

- Age ≥18 years
- Those presented in the ED with pain, discomfort, or pressure in the chest, epigastrium, neck, jaw, or upper limb without an apparent noncardiac source that the treating physician judged needed further assessment due to the probability of ACS
- Blood samples extracted for POC-cTnI assay and hs-cTnI simultaneously

### 2.3. Exclusion Criteria

- Age <18 years.
- Unable to provide informed consent
- Unwilling to participate
- Their onset of symptoms occurred more than 12 hours before their ED visit
- Evidence of noncardiac source
- With another medical condition requiring hospitalization

### 2.4. Assessments

The American Heart Association's (AHA) definition of AMI, which is a rise or decline in troponin as a change of 30% or higher than the baseline measurement, was used in this study. AMI was diagnosed in a clinical setting consisting of myocardial ischemia that was a rise or fall in troponin and at least one troponin value over the 99th percentile (0.04 ng/mL) in association with symptoms of myocardial ischemia, electrocardiogram (ECG) abnormalities, or imaging evidence of fresh loss of viable myocardium. For the diagnosis of AMI, central laboratory cTnI levels were assessed at the time of presentation (0 hour) and reassessed at least 3 hours later. The POC-cTnI was measured at 0 hour in all patients and at 3 hours if a patient was diagnosed with AMI but had a 0-hour negative result for the POC-cTnI assay.

A homogenous, sandwich chemiluminescent technique with a reference range of 0.00–0.04 ng/mL was automatically measured as the central laboratory cTnI (ADVIA Centaur TnI-Ultra assay, Siemens, Germany) on 100 *μ*L serum, heparinized plasma, or ethylenediaminetetraacetic acid plasma sample. In this technique, human cTnI binds to anti-cTnI antibodies (polyclonal goat anti-cTnI antibody labelled with acridinium ester and two biotinylated mouse monoclonal anti-cTnI antibodies; binary lite reagents), which promotes a reaction to produce a chemiluminescent signal proportional to the sample's cTnI concentration. All the reagents (i.e., binary lite reagents and solid phase [latex magnetic particles in buffer with stabilizers and preservatives]) are contained within the ReadyPack reagent pack. The system automatically performs the actions.

The Instant-View POC-cTnI qualitative assay (INSTANT-VIEW TM Troponin I Whole Blood/Serum Test [Cassette], USA) was used as the rapid chromatographic membrane-based immunoassay. It was applied on fresh (nonhemolyzed) serum, plasma, fingerstick, or whole venipuncture blood, enabling quick cTnI qualitative measurement in about 10 minutes.

In this test, a specimen (serum or plasma [1 drop, ∼50 *μ*L] or whole blood [∼70 *μ*L]) is added to the specimen area of the cassette. Capture reagent (0.03% ProClin 300, 1 drop of buffer) is immobilized in the test line region of the cassette. The specimen reacts with anti-cTnI antibody-coated colloid gold particles in the test. This mixture migrates chromatographically along the test length and interacts with the capture reagent. If the specimen contains cTnI, a color line will appear in the test region, reflecting the positive result. A fixed control colored line indicates that proper specimen volume has been added and membrane wicking has occurred (correct procedural technique). Worth noting, the intensity of the color in the test region will vary depending on the concentration of cTnI present in the specimen. Therefore, any shade of color in the test line should be considered positive.

According to the manufacturer's instruction, the detection limit for our center cTnI assay (Bayer ADVIA Centaur) is 0.002 ng/mL, with the 99th percentile cut-off point at 0.04 ng/mL, and according to the manufacturer's instruction, the detection limit for the Instant-View POC-cTnI qualitative assay measured by Bayer ACS:180 is 0.1 ng/ml.

We perform routine quality control of measurements for both the POC-cTnI and hs-cTnI assays. Quality control material was run at the beginning of each shift after an instrument was serviced, when reagent lots were changed, after calibration, and when patient results seemed inappropriate. For POC-cTnI, we repeated the test with the new kit when the control line was negative. We avoid using hemolyzed, icteric, and lipemia samples as these samples may affect the test result [12].

Finally, a follow-up on the 30^th^ day of presentation through telephone contact and patient's notes review was scheduled to assess MACE, including AMI, cardiac mortality, cardiogenic shock, emergency revascularization, ventricular arrhythmia, or high degree of heart block that prompted urgent therapy.

### 2.5. Data Collection

During symptoms and if asked by medical staff, electrocardiograms were recorded at presentation and 6 hours later. The attending clinician made the decision to do stress testing, coronary angiography, and other care strategies. Basic demographics, smoking history, comorbid diseases, family history of ACS, physical findings, time from onset of symptoms to arrival in the ED, and ECG results were all obtained. The final diagnosis was based on the hs-cTnT results and all available clinical and imaging results, electrocardiogram, and routine laboratory testing according to the American Heart Association's (AHA) definition of AMI [11]. All NSTEMI cases were confirmed by coronary angiography (CAG), and expert cardiologists determined the final diagnosis of all patient conditions. Patients who were discharged from the ED were tracked for a month via their clinical information system to see if they needed to return to the ED with cardiac symptoms. The goal of the research was to see how accurate the Instant-View POC-cTnI was at diagnosing ACS in patients with chest discomfort.

### 2.6. Statistical Analysis

Statistical analysis was carried out using SPSS software (version 26.0; IBM Corp., Armonk, NY, USA). Qualitative and quantitative variables were described using frequency (percentage) and median (interquartile range [IQR]) and visualized using bar charts. This study evaluated the sensitivity, specificity, positive predictive value, and negative predictive value (NPV) with a 95% confidence interval (95% CI) to assess the diagnostic accuracy of the Instant-View POC-cTnI qualitative assay according to the central laboratory cTnI results, as the reference.

## 3. Results

### 3.1. Basic Data of Participants

A total of 180 patients were examined. The 30-day follow-up period for these participants began on the initial presentation, and subsequent enrolments were removed from the study. There were 136 individuals left for qualitative POC-cTnI and hs-cTnI assays. The median age of the participants was 59.5 (IQR: 21) years. The majority of the patients (*n* = 77, 57.5%) were male (median age of 58 years [IQR: 16.5]), and 56 patients (41.8%) were female (median age of 60 years [IQR: 26]). Gender was missing for three participants.

### 3.2. Test Performance Results of POC-cTnI Qualitative Assay for Diagnosis of AMI According to Serial hs-cTnI Assays

In 86 (63.24%) subjects, hs-cTnI was positive (either initial or serial); nevertheless, AMI was diagnosed in 85 patients according to the AHA's definition of AMI. The final diagnosis of these patients was STEMI (developed new ECG changes while present in the ED) in 23 patients (27.1%) and NSTEMI in 62 patients (72.9%). Coronary angiography was done in all NSTEMI patients and confirmed the diagnosis. One patient had positive troponin without ECG change; however, polymerase chain reaction (PCR) for coronavirus disease 2019 (COVID-19) was positive in that patient.

**Figure 1:**
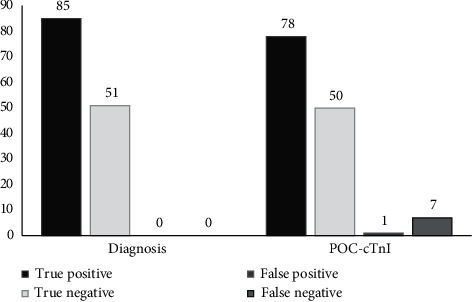
Test performance of 0-hour instant-view POC-cTnI qualitative assay for diagnosis of AMI according to the serial hs-cTnI assays.

In this study, 50 (36.76%) patients with a negative hs-cTnI test were discharged from the ED straight to their homes. It should be noted that sensitivity of 97.65% (95% CI: 91.76–99.71%) and specificity of 98.04% (95% CI: 89.55–99.95%) were obtained for the 0-hour hs-cTnI (Figure 1). The 0-hour and 3-hour hs-cTnI had a sensitivity of 100 (95% CI: 95.97–100%) and a specificity of 98.04% (95% CI: 89.55–99.95%).

**Figure 2:**
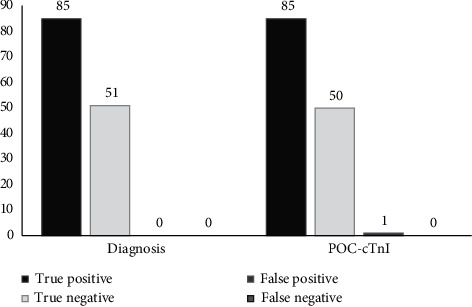
Test performance of 0 and 3 hour instant-view POC-cTnI qualitative assay for diagnosis of AMI according to the serial hs-cTnI assays.

In 85 patients diagnosed with ACS, POC-cTnI was observed to be positive in 78 (sensitivity of 91.76% [95 CI: 83.77–96.62%]) patients at 0 hour and 85 (sensitivity of 100%) patients at 3 hours. In addition, in 51 patients who were not diagnosed with ACS, POC-cTnI was negative in 50 of them (specificity of 98.04% [95 CI: 89.55–99.95%]) at 0 hour and 0 and 3 hours (Figures 1 and 2).

When assessed at 0 hour, hs-cTnI failed to identify two patients (false negative) with AMI, yielding an NPV of 96.15% (95% CI: 86.40–98.99%). In contrast, the new POC-cTnI failed to identify seven patients (false negative) with AMI, yielding an NPV of 87.72% (95% CI: 77.82–93.56%) (Figure 1). However, both hs-cTnI and POC-cTnI assays identify all ACS patients when measured at 0 and 3 hours.

Furthermore, among the seven false-negative cases of 0-hour POC-cTnI, five had a positive 0-hour hs-cTnI; nevertheless, in two of them, both 0-hour POC-cTnI and 0-hour hs-cTnI were falsely negative. It is worth noting that considering both the 0-hour and 3-hour POC-cTnI, all AMI cases were correctly identified, yielding a perfect test performance result. Finally, 0-hour and 0- and 3-hour POC-cTnI achieved total accuracy of 94.12% (95% CI: 88.74–97.43%) and 99.26% (95% CI: 95.97–99.98%), respectively (Figures 1 and 2).

### 3.3. Utility of POC-cTnI Qualitative Assay for Predicting MACE

All 50 patients with negative cTnI results (by 0-hour and 3-hour POC-cTnI and hs-cTnI) were tracked for 30 days, and none experienced at least one MACE. Accordingly, the sensitivity for 30-day MACE was identical for both hs-cTnI and qualitative POC-cTnI (Table 1).

## 4. Discussion

To the best of our knowledge, this has been the first study conducted to assess the diagnostic performance of the Instant-View POC-cTnI qualitative assay according to the hs-cTnI assay in diagnosing AMI and predicting 30-day MACE among AMI suspected patients. It was observed that the 0-hour POC-cTnI qualitative assay had a sensitivity and specificity of 91.76% and 98.04%, respectively. These measurements were 100% and 98.04%, respectively, when measured at 0 and 3 hours. Additionally, none of the patients with negative cTnI results experienced at least one MACE [13]. In line with the results of some studies, POC and the hs-cTnI assays gave false-positive results at both 0 and 3 hours. The COVID-19 PCR was positive in this patient, reflecting the microthrombus in such patients in the setting of a hypercoagulable state [13]. This patient underwent coronary angiography, and no evidence of AMI or ECG changes in favour of ACS was observed for him.

Increased troponin levels are common in COVID-19 patients and are linked to a higher risk of death. Troponin levels measured at the time of admission might aid in risk stratification, particularly in identifying individuals at high risk of mortality when troponin levels are high [13]. The validation of these findings will require high-quality prospective research. Viruses, cytokine-driven cardiac injury, microangiopathy, and unmasked coronary artery disease are possible explanations for this event [14]. The increase in cTnI can be attributed to various factors, including myocarditis and cytokine activity, both of which cause cardiac damage. These pathways are debatable, and none has been proven to be the primary source of elevated cTnI levels [5]. COVID-19 begins as a respiratory infection; nonetheless, it quickly spreads to the cardiovascular system due to an imbalance in the renin-angiotensin-aldosterone pathway caused by angiotensin-converting enzyme 2 depletion. This mechanism, driven by the inflammatory response, endothelial dysfunction, and microvascular damage, might exacerbate the clinical course [13].

At this time, none of these processes has been proven to be the primary cause of troponin increase and/or myocardial injury in COVID-19 patients [13]. The causes of false-positive troponin results, including heterophilic antibodies, human antianimal antibodies, autoantibodies, fibrin, rheumatoid factor, endogenous blood products, such as bilirubin, hemoglobin, and lipidemia, and analytical equipment errors, which are observed in immunoassays, were not identified in the studied cases [15].

Since cTn assays have a wide range of analytical features, each one should be validated individually. Moreover, previously, concerns have been raised that POC-cTn assays might lack the analytical sensitivity of central laboratory assays and have higher degrees of imprecision, which means they have lower overall clinical sensitivity for AMI diagnosis [14]. This might suggest that previous POC-cTn assays failed to detect a higher proportion of individuals with AMI, either overall or in the early stages of symptoms. If extra samples were not sent to the central laboratory, this could impact clinical outcomes [16]. To this end, in a systematic review, no improvement was observed in outcomes, although they were not poorer either. However, it should be emphasized that some of the included studies used older and less sensitive assays [2].

Although POC-cTn does not have the sensitivity of hs-cTn, it can be tested swiftly at the bedside and shorten TAT and therapy time among individuals with AMI [17]. The American College of Cardiology/AHA and the National Academy of Clinical Biochemistry recommend a maximum TAT of 60 minutes for central laboratories. However, the time needed for preparing results by the central hospital laboratory is often delayed by about 1 or more than 2 hours. The shorter TAT of POC assays (15–20 minutes) can make serial sampling over 3 hours possible and potentially speed up clinical decision-making and patient discharge (negative results) with adequate diagnostic accuracy. It can reduce ED visits, be cost-beneficial, and possibly lead to faster treatment paths (positive results) and improve outcomes. The POC-cTnI assay used in the present study has 15–20 minutes of analyser run time and is intended for use at the bedside [18]. The findings of the current study and those of earlier studies demonstrate that the POC system is a suitable test for quick evaluation (reduced TAT and LoS) of ED visits related to ACS symptoms [19].

Only a few studies have compared hs-cTn and newer, possibly more sensitive, POC-cTn assays [7,11]. The results are mixed. However, it appears that more recent studies using newer assays showed that POC-cTn assays are accurate, correlate well with laboratory testing, and are suitable for the rapid evaluation of patients presenting to the ED; nevertheless, none of them had used qualitative POC-cTn assays [19].

This study demonstrated that the 0-hour POC-cTnI qualitative assay had 91.76% and 98% sensitivity and specificity, respectively. Juliano and Wason [20] showed that POC-cTnI at the cut-off value of 0.12 ng/mL yielded the sensitivity and specificity of 100% and 99%, respectively. Body et al. [21] reported that POC-cTnI yielded a sensitivity of 99% and specificity of 98.9%. Similarly, Christ et al. [22] discovered that 3-hour POC-cTnI obtained a sensitivity of 90% and specificity of 96% using cut-off values of cTnI >43 ng/L. Another finding of the current study was a total accuracy of 99.26% for 0-hour and 3-hour POC-cTnI qualitative assays. This finding is comparable with the findings of Peacock et al.'s [23] study, which showed that the total performance values of three different assays (i.e., Alere Triage Cardio3 TnI, PathFast cTnI-II, common central laboratory assay, and Singulex Erenna TnI assay for diagnosing AMI) were 95%, 95%, and 93%, respectively. Furthermore, the sensitivity of 100% and specificity of 98% were obtained for 0-hour and 3-hour POC- cTnI.

Most ED patients need to be assessed for a long time before being safely discharged. This could be accompanied by high healthcare expenses and overcrowding in EDs. Therefore, the correct identification of patients who should be watched for suspected AMI is a serious difficulty [24]. Potentially, the early disposition of patients with signs of AMI to outpatient care is facilitated by an efficient rule-out strategy, which helps reduce the rising crowding of EDs [25]. The present study indicated that a 30-day follow-up of patients with negative POC-cTnI results showed that none had experienced any MACE, which is in concordance with the results of Body et al.'s [21] and Boeddinghaus et al.'s [25] studies reporting that the ruled-out patients had cumulative MACE rates of 0% at 30 days. These findings might imply that the new POC-cTnI assays have clinical performance comparable to that of hs-cTnI assays, allowing for accurate early diagnosis following a single baseline blood sample. This is significant because it indicates that AMI can be reliably ruled out within 15 minutes of a blood draw in the ED for numerous patients.

It takes time for the levels of biomarkers to rise. As a result of early admission, the number of false negative outcomes may rise. In all false negative cases measured at 0 hour by both POC (in 7 cases) and hs-cTnI (in 2 cases), the time from symptom onset to arrival in the ED was less than 3 hours. It seems that the negative results at 0 hour in hs-cTnI (in two patients) were due to an insufficient rise in cTn at that time. For POC-cTnI (in seven patients), it was due to the lack of sensitivity of these assays to detect a very low amount of cTn at that time of measurement. Moreover, all AMI patients could be identified (diagnostic performance reached 100%) when POC-cTnI was measured at 0 and 3 hours. Similarly, Mohammad et al. [26] reported that 3-hour POC-cTnI reassessment improved the on-arrival POC-cTnI lower sensitivity. It is possible to conclude that the measurement of POC-cTnI changes at 0 and 3 hours optimizes AMI diagnosis. Therefore, using a 0-hour + 3-hour blood sampling protocol to identify patients with AMI appropriately is clinically applicable and has promising results in clinical practice, very close to the current guidelines recommendations [9].

One of the limitations of the present study is being performed in a single observational center. In addition, being qualitative, this POC-cTnI assay did not allow to study clinical performance and use quantitative statistical methods, such as Q-Q plot, Bland-Altman analysis, and Passing-Bablok regression. In addition, the sample size was not large enough. As a result, only a small number of ED patients were tested with this new POC-cTnI assay.

Because of the limited sample size, this research should be considered preliminary. This study was done in a relatively high-risk population. This could lead to spectrum bias, which would result in an overestimation of overall test performance. When the POC test is used in other clinical settings, it is important to keep in mind that it may have different characteristics.

Although all laboratory processes followed strict standardized operating protocols, human error in the handling of blood samples may have resulted in inaccurate results in a small number of samples. In addition to the limitations already described, our follow-up time was relatively short. Participants who had an AMI due to a false negative troponin test may not have had an adverse event within a month. Despite these limitations, this study explained the usefulness and limitations of using the measurement of POC cardiac troponins to diagnose ACS in the ED.

## 5. Conclusion

In this small sample-size study, a new qualitative POC-cTnI assay was statistically equal to a hs-cTnI assay in terms of diagnostic accuracy for AMI or MACE in patients with suspected myocardial infarction. The improved clinical sensitivity of this POC-cTnI suggests that such a product is now a viable alternative to central laboratory cTn assays in situations where TAT significantly impacts patient care and speeds up diagnostic procedures in ACS patients in the ED. According to statistical analysis, the POC tests have a strong correlation with laboratory results. This data can help an emergency physician quickly identify the signs of heart damage while also ensuring that the results are reliable without missing any 30-day MACE.

## Figures and Tables

**Table 1:** Comparison of troponin positive and troponin negative patients.

Variable	+cTnI (*n* = 86)	−cTnI (*n* = 50)
Age	63 [18.75]	52.5 [18.5]
Gender		
Male	54 (65.1%)	26 (49%)
Female	29 (34.9%)	27 (51%)
Diabetes		
Negative	43 (71.7%)	37 (74%)
Positive	17 (28.3%)	13 (26%)
Hypertension		
Negative	40 (66.7%)	25 (50)
Positive	20 (33.3%)	25 (50)
Dyslipidemia		
Negative	35 (58.3%)	38 (76%)
Positive	25 (41.7%)	12 (24%)
Smoking		
Negative	33 (55%)	37 (74%)
Positive	27 (45%)	13 (26%)
Family history of ACS		
Negative	24 (40.7%)	41 (82%)
Positive	35 (59.3%)	9 (18%)
Final diagnosis		
STEMI	23 (27.1%)	—
NSTEMI	62 (72.9%)	—
The median time from the onset of symptoms to arrival in the ED (in hours)	4 (1–10)	3.5 (1–12)
Time intervals (hours) from symptom onset		
0–3 h	15 (17.4%)	13 (26%)
9 h	46 (53.4%)	26 (52%)
9–12 h	25 (29.2%)	11 (22%)
MACE		
Negative	74 (86.1%)	50 (100%)
Positive	12 (13.9%)	0

## Data Availability

The data are included in this published article.
